# Five-Year Change in Intraocular Pressure Associated with Changes in Arterial Blood Pressure and Body Mass Index. The Beijing Eye Study

**DOI:** 10.1371/journal.pone.0077180

**Published:** 2013-10-11

**Authors:** Ya Xing Wang, Liang Xu, Xiao Hui Zhang, Qi Sheng You, Liang Zhao, Jost B. Jonas

**Affiliations:** 1 Beijing Institute of Ophthalmology, Beijing Tongren Hospital, Capital University of Medical Science, Beijing, China; 2 Department of Ophthalmology, Faculty of Clinical Medicine Mannheim, University of Heidelberg, Mannheim, Germany; Zhongshan Ophthalmic Center, China

## Abstract

**Purpose:**

To examine a potential association between longitudinal changes in intraocular pressure (IOP), arterial blood pressure and body mass index (BMI) in a population-based setting.

**Methods:**

The longitudinal population-based Beijing Eye Study included 2355 subjects with an age of 45+ years who were examined in 2006 and in 2011. The participants underwent a detailed ophthalmic examination including tonometry and measurement of arterial blood pressure and BMI.

**Results:**

Data on IOP, arterial blood pressure and BMI measured in 2006 and in 2011 were available for 2257 (95.8%) subjects with a mean age of 59.5±9.7 years. The mean change in IOP was −1.25±2.26 mm Hg, mean change in mean blood pressure −7.4±12.1 mmHg, and mean change in BMI was 0.01±2.04 kg/m^2^. In multivariate analysis, the 5-year change in IOP was significantly associated with a higher change in mean blood pressure (*P*<0.001; standardized regression coefficient Beta:0.11; regression coefficient B:0.02; 95% confidence interval (CI):0.01,0.03) after adjusting for younger age (*P*<0.001;Beta:−0.18;B:−0.04;95% CI:−0.05,−0.03), shorter body stature (*P* = 0.002;Beta:−0.06;B:−0.06;95% CI:−0.03,−0.01), thicker central corneal thickness (*P*<0.001;Beta:0.19;B:0.02;95% CI:0.01,0.02), deeper anterior chamber depth (*P* = 0.01;Beta:0.05;B:0.33;95% CI:0.07,0.60), and lower intraocular pressure at baseline (*P*<0.001;Beta:−0.56;B:−0.42;95% CI:−0.45,−0.39). If the analysis included only longitudinal parameters, the change in IOP was significantly associated with a higher change in mean arterial blood pressure (*P*<0.001;Beta:0.10;B:0.02;95% CI:0.01,0.03) and a higher change in body mass index (*P*<0.04;Beta:0.04;B:0.04;95% CI:0.01,0.09).

**Conclusions:**

In the 5-year follow-up of our population-based sample, a change in IOP was associated with a corresponding change in arterial blood pressure and with a corresponding change in body mass index. These longitudinal data support the notion of a physiological relationship between arterial blood pressure, intraocular pressure and body mass index. These findings may be of interest for the discussion of the pathogenesis of glaucomatous optic neuropathy.

## Introduction

Previous cross-sectional studies have suggested that intraocular pressure (IOP), arterial blood pressure and cerebrospinal fluid pressure are associated with each other [1]. Previous population-based investigations such as the Blue Mountains Eye Study, the Beaver Dam Eye Study, the Central India Eye and Medical Study and the Beijing Eye Study had revealed significant associations between IOP and arterial blood pressure measurements [2]–[12]. Other studies reported on associations between lumbar cerebrospinal fluid pressure measurements and body mass index after adjustment for arterial blood pressure [13], [14]. Most of these investigations were cross-sectional analyses, while only few follow-up studies such as the Beaver Dam Eye Study examined the association between IOP and blood pressure longitudinally [2], [14]–[16]. Since cross-sectional investigations cannot prove a temporal causation, and since the association between body mass index and cerebrospinal fluid pressure was not taken into account in all of the preceding studies, we performed this longitudinal investigation to investigate whether changes in IOP are associated with changes in arterial blood pressure and with changes in body mass index. Since the latter is associated with cerebrospinal fluid pressure, and since cerebrospinal fluid pressure has recently been suggested to be associated with glaucoma, the findings of our study may also be interesting for the discussion of glaucomatous optic neuropathy.

## Methods

### Ethics Statement

The Medical Ethics Committee of the Beijing Tongren Hospital approved the study protocol and all participants gave informed written consent, according to the Declaration of Helsinki.

The Beijing Eye Study is a population-based study in Northern China, which was carried out in the urban district of Haidian in the North of Central Beijing and in the village area of Yufa of the Daxing District south of Beijing [17]. The first survey was performed in the year 2001, with 4439 (83.4%) out of 5324 eligible subjects participating. In 2006 the survey was repeated in which 3251 (73.2%) subjects of the 4439 people examined at baseline returned for a follow-up examination, while 143 (3.2%) had died during the follow-up period and 1045 (23.5%) former study participants declined to re-participate. In 2011, the study was again repeated and included 2355 (72.4%) subjects of the 3251 people who were examined in 2006, while 138 (4.2%) subjects had died and 758 (23.3%) former study participants declined to re-participate.

In the surveys, the study participants underwent an interview with standardized questions on their family status, level of education, income, quality of life, psychic depression, physical activity, known major systemic diseases such as arterial hypertension and diabetes mellitus and quality of vision. The ophthalmic examination included measurement of presenting visual acuity and uncorrected visual acuity. Refractive error was assessed by automatic refractometry (Auto Refractometer AR-610, Nidek Co., Ltd, Tokyo, Japan), if uncorreccted visual acuity was lower than 1.0. Best corrected visual acuity was assessed by subjective refractometry. IOP was measured using a non-contact pneumotonometer (CT-60 computerized tonometer, Topcon Ltd., Japan) by an experienced technician. Three measurements were taken, and the mean of the three measurements was used for further statistical analysis. In the surveys of 2006 and 2011, blood pressure was measured with the participant sitting for at least 5 min. The study participants had refrained from smoking and drinking of coffee, tea, or alcohol for at least 3 h. In addition, any exercise was not performed for the last 30 min prior to the blood pressure measurements. A standardized mercury sphygmomanometer was used, and the cuff size was chosen according to the measured circumference of the upper arm. Body height and weight were recorded. Using optical low-coherence reflectometry (Lensstar 900® Optical Biometer, Haag-Streit, 3098 Koeniz, Switzerland), biometry of the right eyes was performed for measurement of the anterior corneal curvature, central corneal thickness, anterior chamber depth, lens thickness and axial length. A slit lamp examination carried out by an ophthalmologist examined the anterior segment of the eyes. The pupil was dilated using tropicamide once or twice, until the pupil diameter was at least 6 mm. Digital photographs of the cornea and lens and retro-illuminated photographs of the lens were taken using the Neitz CT-R camera (Neitz Instruments Co., Tokyo, Japan). Monoscopic photographs of the macula and optic disc were taken using a fundus camera (Type CR6-45NM, Canon Inc. U.S.A.). In all three surveys, the scheme and flow of the different examination steps were similar, so that they were carried out at approximately the same daytime.

Only those subjects from the surveys from 2006 and 2001 with available data on IOP, arterial blood pressure and body mass index were included into the present study. Statistical analysis was performed using a commercially available statistical software package (SPSS for Windows, version 20.0, IBM-SPSS, Chicago, IL). In a first step, we determined the mean values (presented as mean ± standard error for frequencies; as mean ± standard deviation for all other parameters) and medians of the main outcome parameters. In a second step, we performed univariate analyses of the associations between the change in IOP and systemic and ocular other parameters. In a third step, we carried out a multivariate regression analysis with change in IOP as dependent parameter and all those variables as independent parameters for which the *P*-value was ≤0.10 in the univariate analysis. We removed from the multivariate analysis, step-by-step, those parameters that were no longer associated significantly with IOP as dependent parameter, starting with those parameters that showed the least probable association (highest P-value). 95% confidence intervals (CI) were presented. Only one randomly selected eye per subject was taken for statistical analysis.

## Results

Data on IOP, arterial blood pressure and body mass index in 2006 and in 2011 were available for 2257 (95.8% of the 2355 subjects with examinations in 2006 and 2011) with a mean age of 59.5±9.7 years and a mean refractive error of –0.17±2.08 diopters (Table 1). The subjects not fulfilling the entry criteria as compared to the subjects included into this study were significantly older (age: 62.3±10.6 years; *P*<0.001) and more myopic (refractive error: −0.46±2.57 diopters; *P* = 0.03). Both groups did not differ significantly in gender (*P* = 0.21).

**Table 1:** Demographic characteristics of the study population.

Parameter	Mean ± Standard Deviation	Median	Range
Age (Years) (in 2006)	59.5±9.7	58	45–88
Men/Women	960/1297		
Refractive Error (Diopters) (in 2006)	–0.17±2.08	−0.13	−20.00 to +7.25
Axial Length (mm)	23.2±1.1	23.1	18.96–30.88
Central Corneal Thickness			
Anterior Corneal Curvature Radius (mm)	7.61±0.25	7.62	6.82–8.59
Blood Pressure, Diastolic (mm Hg) (in 2006)	78.9±5.84	78	59–115
Blood Pressure, Systolic (mm Hg) (in 2006)	133.3±11.0	131	107–177
Intraocular Pressure (mm Hg) (in 2006)	15.6±3.0	15	6–30

The mean change in IOP was −1.25±2.26 mm Hg (median: −1 mm Hg; range: −12 to +10 mm Hg), mean change in systolic blood pressure was −3.3±17.2 mmHg, diastolic blood pressure −9.4±11.0 mmHg and mean blood pressure −7.4±12.1 mmHg, and the mean change in body mass index was 0.01±2.04 kg/m^2^ (median: 0 kg/m^2^; range: −22.4 to 11.5 kg/m^2^). In absolute terms, the mean change in IOP was 1.96±1.67 mmHg, in systolic blood pressure 13.9±10.7 mmHg, diastolic blood pressure 11.9±8.1 mmHg, mean blood pressure 11.6±8.2 mmHg, and in body mass index 1.47±1.44 kg/m^2^.

In univariate analysis, the change in IOP was significantly associated with the systemic parameters of younger age (*P*<0.001; r = −0.18), female gender (*P* = 0.004), lower body height (*P* = 0.02; r = −0.05), lower systolic (*P*<0.001; r = −0.09) and lower mean (*P*<0.001; r = −0.02) blood pressure, thinner central corneal thickness (*P* = 0.047; r = −0.04), deeper anterior chamber depth (*P*<0.001; r = 0.08), lower IOP (*P*<0.001; r = −0.48), higher change in systolic blood pressure (*P*<0.001; r = 0.09), higher change in diastolic blood pressure (*P*<0.001; r = 0.10), higher change in mean blood pressure (*P*<0.001; r = 0.10), and higher change in body mass index (*P* = 0.01; r = 0.05). The change in IOP was marginally significantly associated with body weight (*P* = 0.06). The change in IOP was not significantly associated with the systemic parameters of body mass index (*P* = 0.59), level of education (*P* = 0.70), diastolic blood pressure (*P* = 0.75), and axial length (*P* = 0.18).

The multivariate analysis included all parameters as independent parameters which were significantly associated with the change in IOP in univariate analysis. After step wise dropping of the parameters change in body mass index (*P* = 0.79), body weight (*P* = 0.79), mean arterial blood pressure (*P* = 0.74), gender (*P* = 0.66), change in systolic blood pressure (*P* = 0.46), change in diastolic blood pressure (*P* = 0.46), and systolic blood pressure (*P* = 0.07), the 5-year change in IOP was eventually significantly associated with younger age (*P*<0.001), shorter body stature (*P* = 0.002), thicker central corneal thickness (*P*<0.001), deeper anterior chamber depth (*P* = 0.01), lower IOP (*P*<0.001), and higher change in mean blood pressure (*P*<0.001) (Table 2).

**Table 2:** Associations between the Change in Intraocular Pressure and Ocular and Systemic Parameters in the Beijing Eye Study 2006/2011 (Multivariate Analysis).

Parameter	*P*-Value	StandardizedRegressionCoefficient Beta	RegressionCoefficient B	95% ConfidenceInterval of B
Age (Years)	<0.001	−0.18	−0.04	−0.05, −0.03
Body Height (cm)	0.002	−0.06	0.02	−0.03, −0.01
Central Corneal Thickness (µm)	<0.001	0.19	0.02	0.01, 0.02
Anterior Chamber Depth (mm)	0.014	0.05	0.33	0.07, 0.60
Intraocular Pressure (mmHg)	<0.001	−0.56	−0.42	−0.45, −0.39
Change in Mean Arterial Blood Pressure (mmHg)	<0.001	0.11	0.02	0.01, 0.03

If the analysis included only the parameters on the 5-year change, the change in IOP was significantly associated with a higher change in mean arterial blood pressure (*P*<0.001; standardized regression coefficient Beta: Beta: 0.10; regression coefficient B: 0.02 (95% CI: 0.01, 0.03) and a higher change in body mass index (*P*<0.04; Beta: 0.04; B: 0.04 (95% CI: 0.01, 0.09).

## Discussion

In our population-based longitudinal study, the 5-year change in IOP was significantly (*P*<0.001) associated with a higher change in mean blood pressure after adjustment for age, body height, central corneal thickness, anterior chamber depth, and IOP. If the analysis included only parameters of the 5-year change, the change in IOP was significantly associated with a higher change in mean arterial blood pressure and a higher change in body mass index. If the body mass index is taken as surrogate for cerebrospinal fluid pressure, these longitudinal data support the notion of a physiological relationship between arterial blood pressure, IOP and cerebrospinal fluid pressure.

The results of our study agree with previous investigations. In the study by Nomura and colleagues on 69,643 Japanese office workers and their family members, systolic blood pressure and body mass index were positively correlated to IOP [2]. In the cross sectional study by Fukuoka and coworkers on 7313 subjects selected as a normal Japanese sample, IOP was positively correlated with systolic blood pressure and body mass index after adjustment for age, corneal radius, central corneal thickness and refractive error [5]. In the Tajimi Study, higher body mass index and higher mean blood pressure were related with IOP [6]. The Los Angeles Latino Eye Study also revealed associations between IOP and higher blood pressure and larger body mass index [7]. In the Singapore Malay Eye Study, age, central corneal thickness and systolic blood pressure were significant determinants of IOP [8]. In the Japanese Kumejima study, Tomoyose and colleagues showed that younger age, higher body mass index, higher systolic blood pressure, were significantly correlated with higher IOP after adjusting for diabetes, thicker central corneal thickness, and steeper corneal curvature [9]. In the recent Chinese Handan Study, IOP was associated with higher blood pressure and higher body mass index, in addition to younger age, female gender, presence of diabetes mellitus, thicker central cornea and higher myopia [18]. Similar results were obtained in other cross-sectional studies such as the Central India Eye and Medical Study and the Liwan Eye Study from Guangzhou/South China [11].

The new finding in our study was that prospectively evaluated changes in IOP were examined for prospectively measured changes in body mass index and arterial blood pressure in a population-based study. The few previous longitudinal studies by McLeod and by Nakano and the Beaver Dam Eye Study were either not population-based or did not simultaneously address arterial blood pressure and body mass index in their associations with IOP after adjustment for other ocular and systemic parameters [14]–[16]. The synopsis of these longitudinal investigations agrees with our findings on associations between higher changes in IOP and higher changes in body mass index and in arterial blood pressure in a longitudinal manner.

Taking body mass index as surrogate for cerebrospinal fluid pressure as suggested in several previous studies [12], [13], the results of our study and of previous investigations suggest a physiologic correlation between arterial blood pressure, cerebrospinal fluid pressure and IOP [1]. Since arterial blood pressure is the highest of the three pressure parameters, one may assume that blood pressure is the driving force behind cerebrospinal fluid pressure and IOP. In a previous study, it appeared that the influence of arterial blood pressure on the cerebrospinal fluid pressure was similar to the influence of arterial blood pressure on IOP, since the trans-lamina cribrosa pressure defined as difference between lumbar cerebrospinal fluid pressure and IOP was not related to arterial blood pressure [1]. The interpretation of the results of our study must take into account that the relationship between arterial blood pressure and cerebrospinal fluid pressure may not hold true in clinical situations with an acutely elevated brain pressure as shown in a study on patients with brain injury [19]. In that study, IOP was related with arterial blood pressure, while cerebrospinal fluid pressure was neither related with arterial blood pressure nor with IOP. In a similar manner, the relationship between IOP, arterial blood pressure and cerebrospinal fluid pressure may not be valid in patients with an acutely elevated IOP such as in angle closure glaucoma. Excluding these situations with acutely altered brain pressure or IOP, the finding of our study may be of interest for the pathogenesis of glaucomatous optic neuropathy which in some previous studies was discussed to reflect an imbalance in the relationships of IOP, cerebrospinal fluid pressure and arterial blood pressure [1], [20]–[23]. Either the IOP is elevated as in conventional high-IOP glaucoma. Or, as assumed in some patients with normal-(intraocular-)pressure glaucoma, a low arterial blood pressure is associated with a more marked reduction in cerebrospinal fluid pressure than in IOP. It results in an increased trans-lamina cribrosa pressure difference, similar as if the IOP is elevated as in high-(intraocular-)pressure glaucoma.

The present investigation is a follow-up of previous studies from the Beijing Eye Study in which among others, the topic of a cross-sectional analysis of the association between body mass index and neuroretinal rim area was examined [24]. That study had revealed that a larger neuroretinal rim area was significantly correlated with a higher body mass index (*P*<0.001), a lower intraocular pressure (*P* = 0.004), lower mean blood pressure (*P* = 0.02), and higher ocular perfusion pressure, after adjustment for optic disc size, presence of open-angle glaucoma, refractive error, age and gender. The findings of the present longitudinal investigation fit with the results of the previous cross-sectional study on the associations between intraocular pressure, body mass index, and arterial blood pressure.

Other previous studies have discussed issues related to body mass index and to glaucoma or glaucoma-related indices [24]–[27]. Pasquale and colleagues assessed the relationship between anthropometric measures and incident primary open-angle glaucoma in 78,777 women of the Nurses' Health Study and in 41,352 men of the Health Professionals Follow-up Study [25]. They found that among women, a higher body mass index was associated with a lower risk of primary open-angle glaucoma with intraocular pressure readings of 21 mmHg or less at diagnosis. In the Barbados Eye Study, persons most likely to have open-angle glaucoma were older men and had a family history of open-angle glaucoma, high intraocular pressure, lean body mass, and cataract history [27]. In a similar manner, Zheng and colleagues in the Singapore Malay Eye Study and Xu and colleagues in the Beijing Eye Study found that persons who were taller or had lower body mass index had a smaller neuroretinal rim area and a larger optic cup-to-disc area ratio [24], [26]. In the Central India Eye and Medical Study, glaucoma prevalence was associated with lower body mass index after adjusting for higher age, lower blood hemoglobin concentration, higher intraocular pressure, disc hemorrhages, higher prevalence of myopic retinopathy, lower level of education, longer axial length, thinner retinal nerve fiber layer, higher vertical cup/disc diameter ratio and narrow anterior chamber angle [28]. In the Japanese Tajimi Study, IOP was associated with higher body mass index after adjusting for younger age, higher mean blood pressure, history of diabetes, thicker cornea, higher myopia and steeper corneal curvature [6]. In a similar manner in the Japanese Kumejima study, higher IOP was significantly correlated with higher body mass index after adjusting for younger age, higher systolic blood pressure, history of diabetes mellitus, thicker central corneal thickness, steeper corneal curvature and longer axial length [29]. Other studies such as an investigation by Gasser and colleagues and a recent longitudinal cohort study by Newman-Casey and colleagues did not find clear associations between body mass index and the prevalence of glaucoma [30], [31]. The latter study included more than 2 million beneficiaries with an age of ≥40 years, who were continuously enrolled in a managed care network and who had 1 or more visits to an eye care provider during the period of 2001 to 2007 [31]. Using billing codes to identify individuals with open-angle glaucoma, the authors found in a multivariable regression model, that obese women as compared with non-obese women had a 6% increased hazard of developing open-angle glaucoma, while obese men as compared with non-obese men had no significant increased hazard of developing open-angle glaucoma OAG. In a similar manner and in contrast to some previous studies, higher body mass index was associated with a thinner retinal nerve fiber layer in men in the large-scaled EPIC-Norfolk Eye Study, which included 11,030 eyes of 6309 participants with mean age 68 years and in which a thinner retinal nerve fiber layer was additionally associated with older age, male gender, shorter axial length and pseudophakia after adjustment for possible confounders [32].

Potential limitations of our study should be mentioned. First, a major concern in any population-based study is nonparticipation. The non-participation of eligible subjects may however have a more marked effect in cross-sectional prevalence studies than in longitudinal investigations such as our study. Second, the measurements of IOP and arterial blood pressure in 2006 and 2011 depended on single measurements. IOP and blood pressure were however measured in a standardized manner at the same time of the day since the study design of the Beijing Eye Study 2006 and 2001 did not differ markedly. It may suggest that variations in diurnal factors physiologically influencing IOP and blood pressure and variations in factors such as agitation and previous caffeine intake may not have markedly influenced the measurements. The alternative for single IOP measurements would have been diurnal pressure curve measurements, however that option may not be feasible for a population-based study. Third, the coefficients for the relationships between the change in IOP and the changes in blood pressure and the in body mass index were relatively low. Although these relationships were statistically significant with a *P*-value of <0.05, the low coefficients showed that only a fraction of the variability in the change in IOP could be explained by that relationship. Strengths of the study were the relatively large study population size and its design as a population-based investigation.

In summary, we demonstrated that in the 5-year follow-up of our population-based sample, a change in IOP was strongly associated with a corresponding change in arterial blood pressure and with a corresponding change in body mass index. These longitudinal data support the notion of a physiological relationship between arterial blood pressure, IOP and body mass index. Since body mass index is correlated with cerebrospinal fluid pressure, future studies may address whether changes in cerebrospinal fluid pressure are associated with changes in IOP and arterial blood pressure, supporting the notion that all three pressure-related parameters are correlated with each other. The findings may be of interest for the discussion of the pathogenesis of glaucomatous optic neuropathy.

## References

[1] Ren R, Jonas JB, Tian G, Zhen Y, Ma K, et al (2010) Cerebrospinal fluid pressure in glaucoma. A prospective study. Ophthalmology 117: 259–266. 19969367 10.1016/j.ophtha.2009.06.058

[2] Nomura H, Shimokata H, Ando F, Miyake Y, Kuzuya F (1999) Age-related changes in intraocular pressure in a large Japanese population: a cross-sectional and longitudinal study. Ophthalmology 106: 2016–2022. 10519601 10.1016/S0161-6420(99)90417-7

[3] Mitchell P, Lee AJ, Wang JJ, Rochtchina E (2005) Intraocular pressure over the clinical range of blood pressure: blue mountains eye study findings. Am J Ophthalmol 140: 131–132. 16038656 10.1016/j.ajo.2004.12.088

[4] Xu L, Wang H, Wang Y, Jonas JB (2007) Intraocular pressure correlated with arterial blood pressure: the Beijing eye study. Am J Ophthalmol 144: 461–462. 17765433 10.1016/j.ajo.2007.05.013

[5] Fukuoka S, Aihara M, Iwase A, Araie M (2008) Intraocular pressure in an ophthalmologically normal Japanese population. Acta Ophthalmol 86: 434–439. 18039350 10.1111/j.1600-0420.2007.01068.x

[6] Kawase K, Tomidokoro A, Araie M, Iwase A, Yamamoto T, et al (2008) Ocular and systemic factors related to intraocular pressure in Japanese adults: the Tajimi study. Br J Ophthalmol 92: 1175–1179. 18669541 10.1136/bjo.2007.128819

[7] Memarzadeh F, Ying-Lai M, Azen SP, Varma R (2008) Los Angeles Latino Eye Study Group (2008) Associations with intraocular pressure in Latinos: the Los Angeles Latino Eye Study. Am J Ophthalmol 146: 69–76. 18486096 10.1016/j.ajo.2008.03.015 PMC2518614

[8] Wong TT, Wong TY, Foster PJ, Crowston JG, Fong CW, et al (2009) The relationship of intraocular pressure with age, systolic blood pressure, and central corneal thickness in an asian population. Invest Ophthalmol Vis Sci 50: 4097–4102. 19458324 10.1167/iovs.08-2822

[9] Tomoyose E, Higa A, Sakai H, Sawaguchi S, Iwase A, et al (2010) Intraocular pressure and related systemic and ocular biometric factors in a population-based study in Japan: the Kumejima study. Am J Ophthalmol 150: 279–286. 20570236 10.1016/j.ajo.2010.03.009

[10] Jonas JB, Nangia V, Matin A, Sinha A, Kulkarni M, et al (2011) Intraocular pressure and associated factors: the central India eye and medical study. J Glaucoma 20: 405–409. 21897285 10.1097/IJG.0b013e3181f7af9b

[11] Wang D, Huang W, Li Y, Zheng Y, Foster PJ, et al (2011) Intraocular pressure, central corneal thickness, and glaucoma in chinese adults: the Liwan eye study. Am J Ophthalmol 152: 454–462.e1. 21679915 10.1016/j.ajo.2011.03.005

[12] Ren R, Wang N, Zhang X, Tian G, Jonas JB (2012) Cerebrospinal fluid pressure correlated with body mass index. Graefes Arch Clin Exp Ophthalmol 250: 445–446. 21814821 10.1007/s00417-011-1746-1

[13] Berdahl JP, Fleischman D, Zaydlarova J, Stinnett S, Allingham RR, et al (2012) Body mass index has a linear relationship with cerebrospinal fluid pressure. Invest Ophthalmol Vis Sci 53: 1422–1427. 22323469 10.1167/iovs.11-8220 PMC3339912

[14] McLeod SD, West SK, Quigley HA, Fozard JL (1990) A longitudinal study of the relationship between intraocular and blood pressures. Invest Ophthalmol Vis Sci 31: 2361–2366. 2243000

[15] Klein BE, Klein R, Knudtson MD (2005) Intraocular pressure and systemic blood pressure: longitudinal perspective: the Beaver Dam Eye Study. Br J Ophthalmol 89: 284–287. 15722304 10.1136/bjo.2004.048710 PMC1772559

[16] Nakano T, Tatemichi M, Miura Y, Sugita M, Kitahara K (2005) Long-term physiologic changes of intraocular pressure: a 10-year longitudinal analysis in young and middle-aged Japanese men. Ophthalmology 112: 609–616. 15808252 10.1016/j.ophtha.2004.10.046

[17] Xu L, Cao WF, Wang YX, Chen CX, Jonas JB (2008) Anterior chamber depth and chamber angle and their associations with ocular and general parameters. The Beijing Eye Study. Am J Ophthalmol 145: 929–936. 18336789 10.1016/j.ajo.2008.01.004

[18] Zhou Q, Liang YB, Wong TY, Yang XH, Lian L, et al (2012) Intraocular pressure and its relationship to ocular and systemic factors in a healthy Chinese rural population: the Handan Eye Study. Ophthalmic Epidemiol 19: 278–284. 22978528 10.3109/09286586.2012.708084

[19] Czarnik T, Gawda R, Kolodziej W, Latka D, Sznajd-Weron K, et al (2009) Associations between intracranial pressure, intraocular pressure and mean arterial pressure in patients with traumatic and non-traumatic brain injuries. Injury 40: 33–39. 19135194 10.1016/j.injury.2008.10.010

[20] Jonas JB, Wang NL (2012) Association between arterial blood pressure, cerebrospinal fluid pressure and intraocular pressure in the pathophysiology of optic nerve head diseases. Clin Exp Ophthalmol 40: e233–234. 21668775 10.1111/j.1442-9071.2011.02625.x

[21] Morgan WH, Yu DY, Cooper RL, Alder VA, Cringle SJ, et al (1995) The influence of cerebrospinal fluid pressure on the lamina cribrosa tissue pressure gradient. Invest Ophthalmol Vis Sci 36: 1163–1172. 7730025

[22] Berdahl JP, Allingham RR, Johnson DH (2008) Cerebrospinal fluid pressure is decreased in primary open-angle glaucoma. Ophthalmology 115: 763–768. 18452762 10.1016/j.ophtha.2008.01.013

[23] Wang N, Xie X, Yang D, Xian J, Li Y, et al (2012) Orbital cerebrospinal fluid space in glaucoma. Ophthalmology 119: 2065–2073.e1. 22749084 10.1016/j.ophtha.2012.03.054

[24] Xu L, Wang YX, Wang S, Jonas JB (2012) Neuroretinal rim area and body mass index. PLoS One 7: e30104. 22253892 10.1371/journal.pone.0030104 PMC3253809

[25] Pasquale LR, Willett WC, Rosner BA, Kang JH (2010) Anthropometric measures and their relation to incident primary open-angle glaucoma. Ophthalmology 117: 1521–1519. 20382429 10.1016/j.ophtha.2009.12.017 PMC2904416

[26] Zheng Y, Cheung CY, Wong TY, Mitchell P, Aung T (2010) Influence of height, weight, and body mass index on optic disc parameters. Invest Ophthalmol Vis Sci 51: 2998–3002. 20071668 10.1167/iovs.09-4470

[27] Leske MC, Connell AM, Wu SY, Hyman LG, Schachat AP (1995) Risk factors for open-angle glaucoma. The Barbados Eye Study. Arch Ophthalmol 113: 918–924. 7605285 10.1001/archopht.1995.01100070092031

[28] Nangia V, Jonas JB, Matin A, Bhojwani K, Sinha A, et al.. (2013) Prevalence and associated factors of glaucoma in rural Central India. The Central India Eye and Medical Study. PLoS One 2013; In Print. 10.1371/journal.pone.0076434 PMC3787001 24098790

[29] Tomoyose E, Higa A, Sakai H, Sawaguchi S, Iwase A, et al (2010) Intraocular pressure and related systemic and ocular biometric factors in a population-based study in Japan: the Kumejima study. Am J Ophthalmol 150: 279–286. 20570236 10.1016/j.ajo.2010.03.009

[30] Gasser P, Stumpfig D, Schotzau A, Ackermann-Liebrich U, Flammer J (1998) Body mass index in glaucoma. J Glaucoma 8: 8–11. 10084268

[31] Newman-Casey PA, Talwar N, Nan B, Musch DC, Stein JD (2011) The relationship between components of metabolic syndrome and open-angle glaucoma. Ophthalmology 118: 1318–1326. 21481477 10.1016/j.ophtha.2010.11.022 PMC3129406

[32] Khawaja AP, Chan MP, Garway-Heath DF, Broadway DC, Luben R, et al (2013) Associations With Retinal Nerve Fiber Layer Measures in the EPIC-Norfolk Eye Study. Invest Ophthalmol Vis Sci 54: 5028–5034. 23821204 10.1167/iovs.13-11971 PMC3726240

